# Chagas disease in Brazil: new challenges and perspectives for old problems

**DOI:** 10.1590/0074-02760240279

**Published:** 2025-07-18

**Authors:** Fred Luciano Neves Santos, Veruska Maia da Costa, Rafaella Albuquerque e Silva

**Affiliations:** 1 Fundação Oswaldo Cruz-Fiocruz, Instituto Gonçalo Moniz, Laboratório Avançado de Saúde Pública, Salvador, BA, Brasil; 2 Fundação Oswaldo Cruz-Fiocruz, Instituto Gonçalo Moniz, Grupo de Pesquisa Interdisciplinar em Biotecnologia e Epidemiologia de Doenças Infecciosas, Salvador, BA, Brasil; 3 Fundação Oswaldo Cruz-Fiocruz, Vice-Presidência de Pesquisa e Coleções Biológicas, Programa de Pesquisa Translacional em Doença de Chagas, Fio-Chagas, Rio de Janeiro, RJ, Brasil; 4 Fundação Oswaldo Cruz-Fiocruz, Núcleo de Epidemiologia e Vigilância em Saúde, Brasília, DF, Brasil; 5 Ministério da Saúde, Secretaria de Vigilância em Saúde, Coordenação-Geral de Vigilância de Zoonoses e Doenças de Transmissão Vetorial, Brasília, DF, Brasil

**Keywords:** chronic Chagas disease, mandatory notification, public health surveillance, disease burden, resource allocation, Chagas disease elimination goals

## Abstract

Mandatory notification of chronic Chagas disease (CD) is vital for improving public health responses in Brazil, where millions are affected. Implemented nationally in 2020 and supported by the “e-SUS Notifica” platform in 2023, this system enables accurate disease burden assessment, early diagnosis, and treatment planning. It facilitates resource allocation and targeted interventions, addressing gaps in surveillance and care. Expanding these efforts and ensuring access to treatment is essential for Brazil’s goal of eliminating CD by 2030.

Chagas disease (CD), a neglected, life-threatening infection caused by the protozoan haemoflagellate *Trypanosoma cruzi*, remains a significant public health challenge. The disease typically begins with an acute phase, often asymptomatic or presenting as a nonspecific, self-limiting febrile illness.^1^ Following this initial phase, most individuals enter a lifelong indeterminate stage without overt symptoms. However, 30-40% of infected individuals eventually progress to a chronic phase marked by severe cardiac, gastrointestinal, or neurological complications.^2^ ^,^ ^3^ According to the World Health Organization (WHO), approximately 5.7 million people in Latin America are infected, leading to 7,500 annual deaths, while an estimated 70.2 million individuals in endemic regions remain at risk of infection.^4^ Climate change and migration have further expanded CD prevalence to non-endemic areas.^5^ In Brazil, 1.1 to 4.6 million people are estimated to be infected, with 25.4 million residing in high-risk areas.^4^ ^,^ ^6^ ^,^ ^7^

Despite being discovered over a century ago, CD persists as a significant global health concern.^8^ Brazil achieved a milestone in 2006 by interrupting *T. cruzi* transmission by the primary vector *Triatoma infestans*. However, controlling other triatomine species remains challenging, as Brazil harbours 64 species with varying vectorial capacities.^9^ Blood donor screening and organ donor screening have mitigated alternative transmission routes.^10^ While congenital transmission has declined in areas with effective vector control,^11^ the emergence of acute cases in northern Brazil, particularly in Pará, underscores the increasing role of oral transmission.^12^

The figure illustrates key historical milestones in the understanding, control, and management of CD in Brazil. It highlights important discoveries, the creation of national programs, regulatory actions, and international initiatives aimed at eliminating the disease as a public health concern. This timeline reflects Brazil’s commitment to combating CD through scientific advancements, public health policies, and international collaborations.

**Figure:**
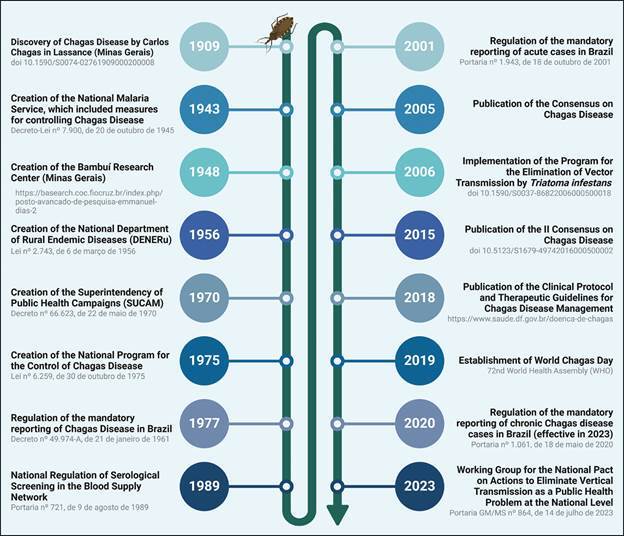
Historical key milestones in the control and management of Chagas disease in Brazil since its discovery.

A pivotal advancement occurred in May 2020 when Brazil revised Regulation No. 4, making chronic CD a nationally notifiable condition in public and private healthcare settings.^13^ Implementation delays caused by the coronavirus disease 19 (COVID-19) pandemic were addressed with the launch of the electronic reporting system, “e-SUS Notifica” in January 2023, positioning Brazil as the sixth country to report chronic CD cases, alongside Chile, Ecuador, Mexico, Paraguay, and Uruguay.^14^ Goiás State had independently adopted chronic CD reporting in 2013, maintaining a historical notification series.^15^

The Brazilian Ministry of Health’s strategy represents a crucial step forward, enabling health authorities to track CD prevalence, assess disease burden, and optimise resource allocation. However, the country’s vast geographic and demographic diversity presents significant challenges. Effective management of chronic CD requires coordinated efforts between health surveillance and primary and secondary healthcare systems. Establishing a comprehensive model of care demands the active involvement of civil society and Health Care Networks to ensure lifelong, continuous care for affected individuals. This includes interventions involving lifestyle modifications, although ensuring continuity of care remains a formidable challenge.

The new health surveillance system also identifies individuals eligible for treatment. Evidence supports the substantial benefits of timely diagnosis and etiological treatment, including prevention of congenital transmission, serological cures in infants and children, and reduced risk of disease progression in treated individuals.^16^ ^,^ ^17^ While Brazil has made significant progress in strengthening CD surveillance and treatment access, experiences from other endemic Latin American countries provide valuable insights. For instance, Argentina and Chile have implemented a national program that integrates congenital CD screening into routine prenatal care, improving early detection and treatment.^18^ Similarly, Bolivia, which has the highest prevalence of CD globally, has expanded mass drug administration efforts and decentralised diagnostic services to enhance case identification and management.^19^ Incorporating lessons learned from these countries could further refine Brazil’s strategy, optimising resource allocation and improving long-term disease control outcomes.

In 2022, Brazil reaffirmed its public health commitment by signing the national pact for the elimination of vertical transmission of HIV, syphilis, hepatitis B, and Chagas disease. This initiative establishes guidelines and targets to reduce vertical transmission of these diseases through testing, treatment, follow-up, and health education, ensuring healthy outcomes for new-borns.^20^

Chronic CD, associated with severe cardiac and gastrointestinal complications, underscores the importance of systematic case reporting. This facilitates early detection and timely treatment, improving outcomes and preventing disease progression.^21^ ^,^ ^22^ ^,^ ^23^ ^,^ ^24^ Accurate case reporting also enhances understanding of CD epidemiology, transmission patterns, risk factors, and long-term consequences, supporting the development of more effective diagnostic tools and treatment strategies. Additionally, the detection of CD cases outside Latin America highlights the importance of robust surveillance to foster international collaboration among health authorities, researchers, and global organisations dedicated to combating the disease.

Despite these advancements, significant challenges remain. Reporting chronic CD cases requires sustained support, monitoring, and active involvement from dedicated teams. In Brazil, health professionals in disease surveillance are responsible for reporting acute and chronic cases. However, reporting alone is insufficient without thorough case investigation and effective treatment access. Identifying individuals with CD but failing to provide antiparasitic therapy undermines surveillance efforts.

A critical challenge is the limited availability of benznidazole, the primary antiparasitic drug in Brazil. Recent shortages have sharply increased prices, exacerbating access issues. Currently, a single manufacturer supplies benznidazole to the Ministry of Health for distribution. To address this bottleneck, alternative manufacturers, such as the Institute of Pharmaceutical Technology (Far-Manguinhos; Oswaldo Cruz Foundation), should be engaged to ensure a stable and affordable drug supply.

Active case finding presents another challenge, as millions of *T. cruzi* carriers remain undiagnosed. Federal, state, and local governments must prioritise epidemiological surveys to identify these individuals. Rapid diagnostic tests (RDTs) offer a promising alternative to resource-intensive diagnostic algorithms. Unlike conventional methods, RDTs require minimal infrastructure and skilled personnel.

The Institute of Immunobiological Technology (Bio-Manguinhos), a division of the Oswaldo Cruz Foundation, has developed the TR Chagas Bio-Manguinhos, a rapid test that surpasses other approved RDTs in Brazil in sensitivity.^25^ Nationwide implementation of this test is underway, with performance evaluations in projects such as Oxente Chagas in Bahia^26^ and Integra Chagas Amazônia in Pará. However, Brazil’s diverse geographic and epidemiological landscape complicates the identification of target populations for serological screening. Given resource constraints, nationwide screening is impractical. Instead, targeted strategies informed by regional and population-specific data are essential for optimising resource allocation and improving detection rates.^7^ ^,^ ^27^

In conclusion, chronic CD reporting is essential for public health surveillance, disease monitoring, treatment delivery, research funding, and fostering international collaboration. It plays a pivotal role in quantifying the disease burden and supporting initiatives to prevent transmission and improve health outcomes. Epidemiological screening should focus on high-risk populations, levering information systems and surveillance tools to monitor vectors.

Achieving the goal of eliminating CD in Brazil by 2030 requires addressing critical challenges, particularly early identification of carriers and their integration into primary healthcare systems across the country’s vast and diverse territory. These efforts will enhance understanding of the affected population and inform targeted interventions, driving progress toward elimination.

As recommendations and future directions, a comprehensive and coordinated strategy is essential to strengthen Brazil’s response to chronic CD. Expanding and enhancing primary healthcare services will facilitate early detection, timely treatment initiation, and long-term disease management. Ensuring a stable and sufficient supply of benznidazole through the engagement of additional pharmaceutical manufacturers is critical to overcoming current shortages and improving treatment accessibility. Optimising diagnostic strategies requires the large-scale implementation of rapid tests, such as TR-Chagas Bio-Manguinhos, alongside targeted screening based on epidemiological data to enhance case identification. Strengthening surveillance through the e-SUS Notifica system will improve case tracking and resource allocation, reinforcing the public health response. Additionally, increasing community engagement and public awareness campaigns will promote early diagnosis and improve treatment adherence.

Achieving the goal of eliminating CD in Brazil by 2030 hinges on overcoming several critical challenges, most notably the early identification of carriers and their seamless integration into primary healthcare systems. Given Brazil’s vast and diverse territory, these efforts are essential to ensure equitable access to care. Strengthening early detection and healthcare integration will provide a clearer understanding of the affected population, enabling the design of more effective and targeted interventions. By addressing these challenges, Brazil can accelerate progress toward the elimination of CD, reducing its burden and improving health outcomes nationwide.
